# The Mechanism of Human Connectedness in Relationship to Physical Health, Mental Health, and Cognitive Function Among the Elderly in Rural China

**DOI:** 10.3389/fpsyt.2021.702603

**Published:** 2021-09-14

**Authors:** Shicun Xu, Yuanyuan Wang, Hui Yu

**Affiliations:** ^1^Northeast Asian Research Center, Jilin University, Changchun, China; ^2^Department of Population, Resources and Environment, Northeast Asian Studies College, Jilin University, Changchun, China; ^3^China Center for Aging Studies and Social-Economic Development, Jilin University, Changchun, China; ^4^Division of Psychology, Faculty of Health and Life Sciences, De Montfort University, Leicester, United Kingdom

**Keywords:** human connectedness, elderly, cognitive function, physical illness, depression

## Abstract

**Background:** Elderly people face particular challenges in their everyday lives, and these are likely to be caused by physical health, mental health, cognitive function, and lack of maintaining a connection to other people. This study aims to explore a meaningful measure of human connectedness among the elderly in rural China and to examine the extent to which it impacts elderly people's physical and mental health as well as their cognitive function.

**Methods:** Survey data were collected from 483 participants who were aged 60 and older in northeast rural China. The outcome variables included cognitive function, which was assessed by the Short Portable Mental Status Questionnaire; depressive symptoms, which were measured by the 10-item version of the Center for Epidemiologic Studies Depression Scale; and the participants' physical health, which was assessed by a 15-item checklist. On the other hand, human connectedness was constructed by perceived family support, which was measured by the Multidimensional Scale of Perceived Social Support; the sense of community, which was measured by the Brief Sense of Community Scale; and the satisfaction of connectedness with others (i.e., with family members and friends).

**Results:** Structural equation modeling analysis confirms that perceived family support, community feelings, and perceived satisfactory connections with family and friends constitute a sufficient representation of human connectedness. Moreover, human connectedness also significantly predicted one's mental and physical well-being as well as cognitive function (b = 0.11, *SD* = 0.02, β = 0.50, *p* < 0.001; model fitting indexes $X_{\left(16\right)}^{2}$ = 17.27, *p* = 0.368, CFI = 0.998, and RMSEA = 0.013).

**Conclusion:** The present study is the first attempt to explore the latent structure of human connectedness and its positive impact on cognitive function as well as physical and mental health among elderly people. The implications and the importance of fostering a stronger social support network, especially for the aging population, are discussed.

## Introduction

The aging population is one of the most complicated social issues that many societies have to face in the modern era (1). According to the sixth Chinese national census, there were 118.83 million people aged 65 years and older, which counted for 8.87% of the total population, and 50.32% of them lived in rural areas (2). The rapidly increasing aging population in China has attracted research interests (3). It is estimated that a great amount of resources needs to be allocated to address elderly people's physical and mental health problems as well as to compensate for their declined cognitive functions (4). Therefore, it is essential to distinguish those important factors that can help the elderly to maintain their physical and mental health as well as preserve their cognitive functions.

Connecting to others is one of the essential needs for all humans (5), whereas human connectedness can be considered a very broad concept that reflects this basic human need. In a landmark study, House et al. (6) show that the lack of social connection might be as, if not more, detrimental to health than obesity or smoking. However, it may be particularly challenging for elderly people to maintain the level of connectedness with others that they used to have. In the rural areas of China, it may be particularly so as many young people leave their hometowns to seek work opportunities in the big cities. In rural China, it is still popular to believe that it is the filial piety duty of the adult children to take care of their parents and older generations (7). Those elderly who are left behind, therefore, may be subjected to disappointment in addition to loneliness coming from the conflict between beliefs in Chinese filial piety and the absence of their children in their everyday life (8). Previous studies show that the elderly receive limited social support and lack of care suffered from mental problems, such as depression and loneliness (9, 10). Loneliness is demonstrated to be an important factor that undermines general health in the elderly (11). A meta-analysis shows that loneliness is associated with decreased cognitive function in the elderly (12). Furthermore, a study in the elderly also indicates that loneliness negatively impacts mood, especially for those with cognitive impairment (13). That is, those factors appear to interact with each other, and the decrease of human connectedness appears to have detrimental impacts on cognition, emotional feelings, and the physical health of the elderly (11).

Currently, there is an absence of study focusing on the association between human connectedness, mental and physical health, and cognitive functions in the elderly in rural China. As such, understanding the perspective and need for connection from these elderly people living in the rural area is the first crucial step to help them adapt to a rapidly changing environment. In a collectivism culture, family is always in the center of one's social life (14). For example, research shows that elderly people with social support are more likely to engage in physical activities, especially when they receive support from family members (15). In addition, a sense of community feeling is also of ultimate importance in the concept of human connectedness as well as being associated with an individual's function and well-being (16).

In the current study, we aimed to investigate the benefits of human connectedness and how human connectedness can be operationalized. We hypothesize that (1) perceived family support, the sense of community, and the direct measure of one's satisfaction with family and friend connectedness is a good starting point in capturing human connectedness among the elderly in rural China and (2) human connectedness has a significant impact on one's cognitive function as well as physical and mental health.

## Materials and Methods

### Participants

The sample consists of 483 participants from Dongliao County, Liaoyuan City, Jilin Province in northeast China. The inclusion criteria is (1) age 60 years and older, (2) having lived in the village for at least 180 days in the past 12 months, and (3) having a local household registration (rural Hu Kou status). Ethical approval for the study was granted by the Ethics Committee of the University of Hong Kong. Sixteen villages out of 235 villages in Dongliao County were randomly chosen, and about 30 participants from each village were recruited based on referrals from the village commissions. There were 241 males and 228 females in the sample. The mean age of the participants was 69.40 (*SD* = 6.21). Other socio-demographic characters of the sample are shown in Table 1.

**Table 1:** Socio-demographic characters of the sample.

**Measure variable**	***n***	**(%)**	**Mean**	**(*SD*)**
**Age**			69.40	(6.21)
60–69	278	(57.56%)		
>70	192	(39.75%)		
Missing data	13	(2.69%)		
**Gender**				
Male	241	(49.90%)		
Female	228	(47.20%)		
Missing data	14	(2.90%)		
**Marital status**				
Never married	3	(0.62%)		
Separation/Divorced	3	(0.62%)		
Widow/Widower	132	(27.33%)		
Married	328	(67.91%)		
Others/missing data	17	(3.52%)		
**Education level**				
<9 years	367	(75.98%)		
9–12 years	96	(19.88%)		
>12 years	3	(0.62%)		
Missing data	17	(3.52%)		
**Cognitive test**			8.84	(1.56)
1–2 errors	411	(85.09%)		
3–4 errors	44	(9.11%)		
>4 errors	17	(3.52%)		
Missing data	11	(2.28%)		
**Physical illness**			1.86	(1.74)
Free from any illness	101	(20.91%)		
Suffering from some illness	361	(74.74%)		
Missing data	21	(4.35%)		
**Depressive symptoms**			6.95	(6.11)
Total score 0–15	415	(85.92%)		
Total score >16	52	(10.77%)		
Missing data	16	(3.31%)		

*SD, standard deviation*.

### Measures

#### Cognitive Function

The Short Portable Mental Status Questionnaire (17) was used to assess participants' cognitive function. Some of the questions were adapted to the Chinese context; for example, “Who is the current president of P. R. China?” and “What day is the Autumn Festival?” Other items of pure cognitive function were directly translated, such as “Count backward from 20 by threes.” Participants' responses were rated as correct (score 1) or incorrect/do not know (score 0). The total score of the cognitive function was calculated ranging from 1 to 10 with a higher score indicating a higher cognitive function. The total score was used in the modeling analysis as a continuous variable. In addition, high or low cognitive function was categorized following the established cutoff scores (18, 19); having fewer than one or two errors (total score ≥8) in their answers is considered normal cognitive functioning, three of four errors considered mild cognitive impairment; five to seven errors moderate cognitive impairment, and eight or more errors severe cognitive impairment. The categorization was used in the summary of the variable only (see Table 1).

#### Physical Illness

Participants were asked whether they suffered from a range of 15 commonly observed conditions among the elderly, including high blood pressure, diabetes, liver disease, etc., and participants chose from “0” (no) or “1” (yes). This measure was adapted from the China Health and Retirement Longitudinal Study (CHARLS) (20, 21). A composite score of physical illness was calculated by adding the responses from the 15 items, ranging from 0 to 15, with a higher score indicating poorer physical health.

#### Depressive Symptoms

Depressive symptoms were measured by a 10-item version of the Center for Epidemiologic Studies Depression Scale (CES-D-10) (22). Participants were asked to rate the frequency of symptoms that they experience (such as “I feel upset”) during the last week on a 0–3 Likert scale with 0 as “almost never” and 3 as “almost every day.” The internal consistency of this measure is high with a Cronbach's alpha of 0.85. The total score was calculated, ranging from 0 to 30, with a higher score indicating a greater level of depressive feeling. The total score was used in the modeling analysis as a continuous variable. Summary information was made according to the suggested cutoff score (23): a total score >16 is considered showing depressive symptoms.

#### Human Connectedness Variables

Human connectedness is conceptualized as receiving good family support, having a warm community feeling, and feeling satisfied with their connectedness to family members and friends.

#### Perceived Family Support

Perceived family support was measured by four items, taken and adapted from the Multidimensional Scale of Perceived Social Support (24) including (1) my family really tries to help me, (2) I receive emotional help and support from my family, (3) I can talk about my problems with my family, and (4) my family helps me to make important decisions. Participants were asked to indicate how much they agree with the statements on a Likert scale with 1 as “strongly disagree” to 5 as “strongly agree.” The internal consistency of this measure is high with a Cronbach's alpha of 0.88. The total score was calculated, ranging from 4 to 20 with a higher score indicating higher perceived family support.

#### The Community Feeling

This measure is based on essential elements from the Brief Sense of Community Scale (25), but the items and the language were adapted to a Chinese context. The community feeling was measured by five items, including (1) people of different backgrounds in the community get along very well; (2) people respect each other in the community; (3) people help each other in the community; (4) people do not only mind their own business, but also take care of others in the community; (5) the community is like a family, and I am one of the contributing members. Participants were asked to indicate how much they agree with the statements on a Likert scale with 1 as “strongly disagree” to 5 as “strongly agree.” The internal consistency of this measure is high with a Cronbach's alpha of 0.95. The total score was calculated, ranging from 5 to 25 with a higher score indicating higher perceived community support.

#### Satisfaction of the Connectedness With Others

Satisfaction with the connectedness with others was directly measured with three items, including (1) the number and mode of contact with family members, (2) the number and mode of contact with friends, and (3) the quality of communication with children and grandchildren. Participants were asked to indicate how satisfied they were with the abovementioned statements with 1 as very much dissatisfied to 5 as very much satisfied. The internal consistency of this measure is high with a Cronbach's alpha of 0.79. The total score was calculated, ranging from 3 to 15 with a higher score indicating a higher satisfaction of their connectedness with others.

#### Data Analysis

All analyses were carried out using R version 4.0.3 for Mac. The structural equation model (SEM) was conducted using the Lavaan package for R (26), examining the relationship between human connectedness and the outcome variables, including physical and mental health as well as cognitive function with participants' age and gender controlled. Model fit indexes, including the chi-square test, the comparative fit index (CFI), and the root mean square error of approximation (RMSEA), were used to assess the goodness of the model fit. It is commonly considered that a non-significant chi-square test, a CFI higher than 0.95, and an RMSEA smaller than 0.05 indicate good model fit. A *p* < 0.05 was considered statistically significant in this study.

## Results

### Descriptive Statistics

Table 1 summarizes the characteristics of the sample. Means, SDs, and the correlations among the study variables are summarized in Table 2. It is worth noticing that this sample has a relatively low level of education with 367 participants (75.98%) spending <9 years in formal schooling. On the other hand, for marital status, never being married or separated/divorced was rare with three participants (0.62%) in each of the abovementioned categories. It is also worth noticing that the participants, on average, did not suffer from a lot of physical illness with *M* = 1.86/15 (*SD* = 1.74). Within the sample, 101 participants (20.91%) reported being free from suffering from any physical illness. In addition, the average cognitive function score (*M* = 8.84, *SD* = 1.56) was in the normal mental functioning range. Among all participants, 411 (85.09%) scored eight or above, indicating normal cognitive function; 44 (9.11%) made three or four errors, indicating mild cognitive impairment; 17 participants (3.52%) made more than four errors, indicating a moderate-to-severe cognitive impairment. Indicated by the CES-D-10 score, 52 participants (10.77%) scored higher than 16/30, indicating that they probably show depressive symptoms. Among the testing variables, except for a non-significant correlation between cognitive function and community feeling, all other pairs were significantly correlated with *p* < 0.001.

**Table 2:** Means, SDs, and bivariate correlation among the study variables.

				**Bivariate correlation**				
		**Mean**	***SD***	**1**	**2**	**3**	**4**	**5**
1	Physical illness	1.86	1.74	–				
2	Depressive symptoms	6.95	6.11	0.37^***^	–			
3	Cognitive function	8.84	1.56	−0.16^***^	−0.26^***^	–		
4	Family support	17.59	2.26	−0.23^***^	−0.23^***^	0.20^***^	–	
5	Community feeling	22.17	2.56	−0.21^***^	−0.18^***^	0.09	0.62^***^	–
6	Satisfaction of connectedness	12.64	1.86	−0.20^***^	−0.34^***^	0.20^***^	0.60^***^	0.51^***^

*SD, standard deviation*;

*** *p < 0.001*.

### Structure Equation Modeling

**Figure 1:**
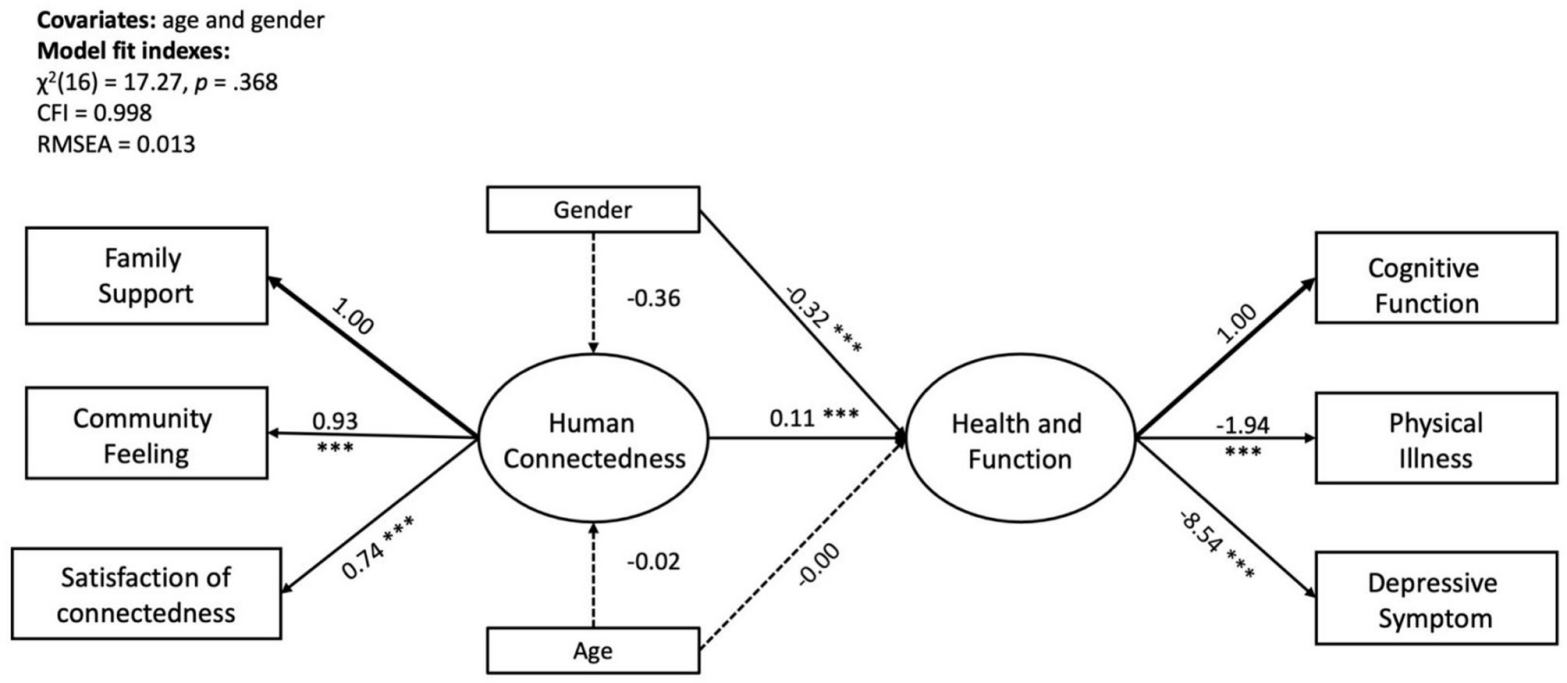
SEM model of human connectedness and health and function with gender and age as covariates. Non-standardized coefficients were reported in the figure; ***p < 0.001.

An SEM analysis was conducted to test the effect of human connectedness on the health and function outcome variables with age and gender being controlled as covariates (Figure 1). The path estimates are shown in Table 3. The model fitting indexes all indicate an adequate model fit with $X_{\left(16\right)}^{2}$ = 17.27, *p* = 0.368, CFI = 0.998, and RMSEA = 0.013. The standardized factor loading estimates for human connectedness range from 0.70 to 0.86. In addition, human connectedness is significantly associated with cognitive function as well as physical and mental health (b = 0.11, *SD* = 0.02, β = 0.50, *p* < 0.001). Among the covariates' paths, gender has a significant effect on the outcome variable construct (b = −0.32, *SD* = 0.07, β = −0.36, *p* < 0.001) as females show significantly lower levels of cognitive function and health. In addition, the variance explained by the model for the three dependent variables is as follows: $R_{\text{cognitive}-\text{function}}^{2}$ = 0.14; $R_{\text{depressive}-\text{symptom}}^{2}$ = 0.38; $R_{\text{physical}-\text{illness}}^{2}$ = 0.26; and for the construct of “health and function,” *R*^2^ = 0.42.

**Table 3:** SEM model summary.

**Factor/Path**	**Estimate**	***SD***	**95% CI**	**Standardized estimate**	***p***
**Human connectedness**					
Family support	1.00			0.86	
Community feeling	0.93	0.11	[0.71, 1.14]	0.70	** <0.001**
Satisfaction with connectedness	0.74	0.05	[0.64, 0.84]	0.78	** <0.001**
**Health and function**					
Cognitive function	1.00			0.37	
Physical illness	−1.94	0.40	[−2.72, −1.16]	−0.51	** <0.001**
Depressive symptoms	−8.54	1.62	[−11.72, −5.36]	−0.62	** <0.001**
**Predictive path**					
Connectedness -> Health and function	0.11	0.02	[0.07, 0.15]	0.50	** <0.001**
**Covariates**					
Gender -> Health and function	−0.32	0.07	[−0.46, −0.18]	−0.37	** <0.001**
Age -> Health and function	0.00	0.00	[−0.03, 0.01]	−0.03	0.632
Gender -> Human connectedness	−0.36	0.20	[−0.75, 0.03]	−0.09	0.069
Age -> Human connectedness	−0.02	0.02	[−0.06, 0.02]	−0.07	0.190

*SD, standard deviation. The bold values indicate that the coefficients are statistically significant*.

## Discussion

The present study is one of the first attempts to explore the latent structure of human connectedness and its positive impact on cognitive function as well as physical and mental health among the elderly in rural China. The results support both of our hypotheses. Perceived family support, community feeling, and the direct measure of one's satisfaction on connectedness are closely correlated constructs, and they form a good measurement model for human connectedness. In addition, human connectedness has a significant impact on the outcome variables, which indicate that human connectedness can be considered a powerful protective factor against the decline of cognitive function and physical as well as mental health among the elderly.

Although human connectedness is not a new concept, there has not been an established measure for this important construct. In the current study, we explored combining family support, community feeling, and a direct measure of one's satisfaction on connectedness to capture the essential elements of human connectedness. These indicators prove forming a good operationalization for human connectedness among the elderly people in rural China. Family support is undoubtedly situated at the center of one's connectedness, which is the hub for individuals to turn to in times of both joy and distress. In Chinese tradition, elderly people rely on their children for care and support; in particular, the elderly son, together with his wife and children, is responsible for taking care of his aging parents (27). However, it is reported that family support for the elderly is weakening in rural China due to rapid societal changes (28). For example, many young people are moving to big cities, leaving their children to the care of their grandparents in rural areas. This change not only deprives the elderly of the expected support of their adult children, it also adds additional responsibility to care for the grandchildren (29). As emphasized by a previous study (11), it is important for healthcare professionals to be alert to the loneliness experience of the elderly and pay attention to the adverse health outcomes of a lack of human connectedness.

Conversely, it is worth noticing that the sample in this study reports a relatively high score of community feeling (mean score of 22.17 out of the maximum score of 25), which was more or less expected in the context of the rural area in northeast China, where people traditionally were closely connected. The model in the current study suggests that community feeling incorporates well into the construct of human connectedness. The sense of community feelings are studied in a wide range of contexts, for example, at workplaces (30) and among adolescents (31) as well as in immigrant communities (32). Existing literature suggests that participating actively in their community affairs and a sense of belonging are positively associated with one's overall health (33). Consistent with findings from some previous studies (34), the findings of this study suggest that the latent construct of human connectedness is associated with higher cognitive function, fewer physical illness conditions, and a lower level of depressive feelings among the elderly in rural China.

Among the outcome variables, the current study shows that 74.74% of the elderly participants suffered from one or more physical illness conditions, 12.63% from mild-to-severe levels of cognitive impairment, and 10.77% showed depressive symptoms. It is worth noticing that the measures used were screening tools rather than rigorous diagnostic tests; also, the smallness of the sample size and the rigor of the sampling method could not support this as a representative sample of the elderly in rural China. Still, our result does signal a strong need for attention to the physical and mental health of elderly people living in rural areas. Previous research also shows that elderly people in rural areas have fewer social resources compared with those in other areas (35). In addition, the current pension system in rural China is extremely insufficient (3), which cannot provide elderly people in rural areas with financial support and security. Combining these results, we see the urgency of providing structural support for elderly people in rural China.

Another interesting finding is that we identified a health disparity in gender comparison with elderly females being at a disadvantage compared with elderly males, which is inconsistent with previous studies in elderly populations in rural China (3, 36). That is, it is important to focus on the elderly population in Chinese rural areas, and specific attention should be given to the more disadvantaged elderly female population. More importantly, females have a longer life expectancy than males, and the disadvantages of elderly females implies that long-term care services should be considered for them (37). Together with the findings in the current study, it is fair to claim that building a stronger social support network focusing on strengthening human connectedness is of ultimate importance for the future of a thriving society.

The current study has some limitations. First, this is a cross-sectional survey; thus, the causality in the measured variables cannot be granted. Future longitudinal study is required to further investigate the mechanism among human connectedness, physical health, mental health, and cognitive function in elderly people. Second, the current sample was recruited from the economically less developed northeast China, which may not represent the other economically developed areas in China. Research indicates that the elderly in urban areas have better living conditions and access to various facilities (38), and it is likely that the elderly in the urban and economically developed regions have better general health. In addition, the sample size was not enough to represent the surveyed economic less developed region. Finally, due to the insufficient resources, we did not offer follow up assessments for participants identified with poor physical, cognitive, and mental health.

## Conclusions

It is with little doubt that, knowing elderly people's needs, fostering and restoring the culture to respect and support the elderly would be of great importance for the entire society to thrive. In conclusion, an improved support system with human connectedness at the center is greatly needed to help the elderly living in rural China cope with the rapidly changing environment in addition to their naturally declining physical and mental health as well as cognitive functioning.

## Data Availability Statement

The data that support the findings of this study are available from the corresponding author (SX), upon reasonable request.

## Ethics Statement

The studies involving human participants were reviewed and approved by Hong Kong University. The patients/participants provided their written informed consent to participate in this study.

## Author Contributions

SX: study design, data collection, manuscript writing, data analysis, and interpretation. HY: data analysis and interpretation, tables and figures editing, and manuscript writing. YW: literature research, data interpretation, and manuscript writing. All authors reviewed and agreed with the final version of the manuscript.

## Conflict of Interest

The authors declare that the research was conducted in the absence of any commercial or financial relationships that could be construed as a potential conflict of interest.

## Publisher's Note

All claims expressed in this article are solely those of the authors and do not necessarily represent those of their affiliated organizations, or those of the publisher, the editors and the reviewers. Any product that may be evaluated in this article, or claim that may be made by its manufacturer, is not guaranteed or endorsed by the publisher.
